# Research Progress on the Flexibility of an Implantable Neural Microelectrode

**DOI:** 10.3390/mi13030386

**Published:** 2022-02-28

**Authors:** Huiqing Zhao, Ruping Liu, Huiling Zhang, Peng Cao, Zilong Liu, Ye Li

**Affiliations:** 1 Beijing Institute of Graphic Communication, Beijing 102600, China; zhaohuiqing2020@gmail.com (H.Z.); liuruping@bigc.edu.cn (R.L.); zhanghuiling429@gmail.com (H.Z.); pc@bigc.edu.cn (P.C.); 2 Division of Optics, National Institute of Metrology, Beijing 100029, China; liuzl@nim.ac.cn

**Keywords:** neural microelectrode, flexible materials, electrode structure, electrode implantation method

## Abstract

Neural microelectrode is the important bridge of information exchange between the human body and machines. By recording and transmitting nerve signals with electrodes, people can control the external machines. At the same time, using electrodes to electrically stimulate nerve tissue, people with long-term brain diseases will be safely and reliably treated. Young’s modulus of the traditional rigid electrode probe is not matched well with that of biological tissue, and tissue immune rejection is easy to generate, resulting in the electrode not being able to achieve long-term safety and reliable working. In recent years, the choice of flexible materials and design of electrode structures can achieve modulus matching between electrode and biological tissue, and tissue damage is decreased. This review discusses nerve microelectrodes based on flexible electrode materials and substrate materials. Simultaneously, different structural designs of neural microelectrodes are reviewed. However, flexible electrode probes are difficult to implant into the brain. Only with the aid of certain auxiliary devices, can the implant be safe and reliable. The implantation method of the nerve microelectrode is also reviewed.

## 1. Introduction

Since ancient times, brain diseases have been a major problem threatening human’s life, such as epilepsy, amyotrophic lateral sclerosis, dementia, and stroke [1]. Conventional treatment methods including drug therapy and neurosurgery have many side effects. In recent years, a new generation of disease treatment methods based on neural microelectrode array have developed rapidly, providing a more safe and reliable treatment for brain diseases. At present, electrode arrays such as cochlear implant for hearing restoration and visual prosthesis for vision reconstruction are applied [2,3]. As the most important part of neural engineering system, the electrode plays an important role in the interface between the nerve and electron. Its function is mainly manifested in two forms: one is recording the electrical signal of the nerve activity, and the other is utilizing the current to stimulate and inhibit nerve activity to achieve functional stimulation, ultimately achieve the role of neuropathy treatment [4,5].

The first implanted electrode for recording brain activity over a long time was made of insulated microwires [6]. Inside the microwire electrode is a metal electrode, which is wrapped in an insulating material and has an opening at the tip for recording signals [7]. However, due to the lack of rigidity, easy bending, and deformation, it cannot reach the predetermined implantation area. Additionally, in the preparation process, the manual operation part is very large, and it is difficult to achieve the consistency of the electrode. Compared with metal microwire electrodes, electrode arrays based on silicon have a higher mechanical hardness and biocompatibility. The most representative silicon-based electrodes include Michigan electrodes and Utah electrodes [8,9]. Hochberg et al. [10] implanted a silicon-based microelectrode array into the motor cortex of male patients who had been paralyzed for three years, and recorded the change trend of the nerve electrode signal for nine months. The electrical signal is transmitted to the external device, so that the patient can control the TV, cursor movement on the screen, and other behaviors through the nerve, which makes a great contribution to the recovery of normal life of the paralyzed patients. However, due to the high rigidity of silicon-based materials (Young’s modulus of single-crystal silicon is ~170 GPa, and that of brain tissue is ~3 kPa), they cannot match the biological tissue. Furthermore, most of the biological tissues have curved surfaces, so it is difficult for silicon-based electrodes to achieve a close connection with the nerve tissues of the epicortical. The micro movement of the tissue will also trigger immune reactions [11,12]. 

**Figure 1:**
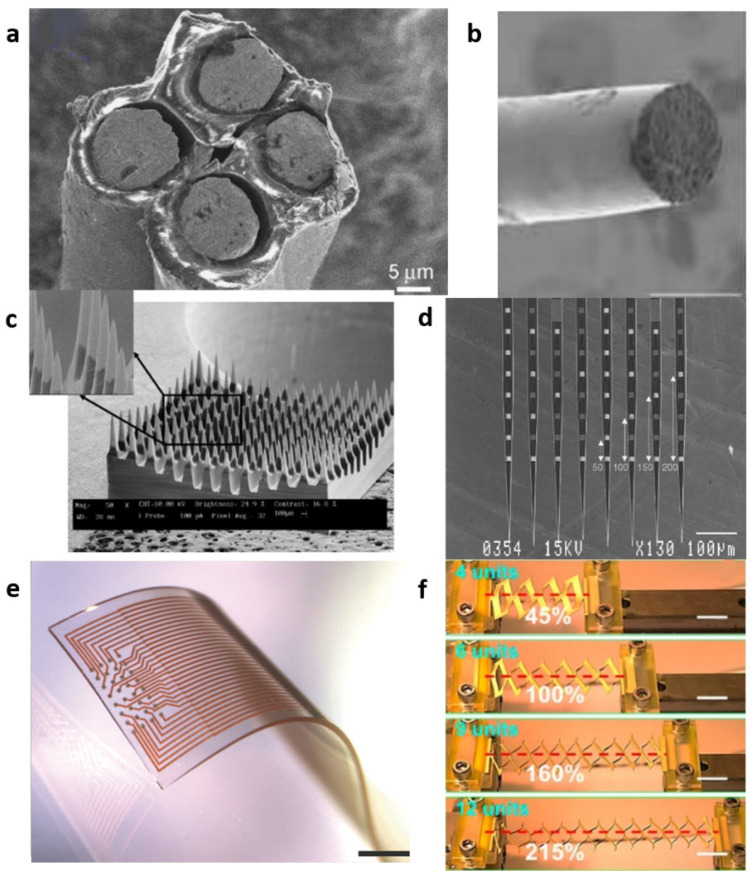
Types of neural electrode: (**a**) tetrode [23], (**b**)single wire [24], (**c**) Utah electrode [25], (**d**) Michigan electrode [26], (**e**) high-density stretchable electrode grids for chronic neural recording [27], and (**f**) kirigami [28].

Recently, the implantable flexible nerve microelectrode made of flexible and stretchable materials can solve the injury of the nerve tissue and the occurrence of immune rejection, making the electrode record neuron signals and stimulate nerve cells safely and reliably for a long time [13]. Young’s moduli of traditional materials (silicon, glass, and metal) range from 50 to 200 GPa, orders of magnitude higher than those of the nervous tissues, which are typically 3.15–10 kPa [14]. The implantable flexible nerve microelectrode is mainly composed of a substrate, an electrode, and a package. For example, materials such as perylene-C, polyamide, and SU-8 have a low Young’s modulus of 1–10 GPa and are employed as the substrate material for the neural microelectrode. PDMS can achieve an even lower Young’s modulus of 1 MPa, becoming one of the softest substrate materials for the nerve microelectrode [15]. In addition, structural design, such as the wave, serpentine, origami, and kirigami designs, is an effective strategy for electronics with flexibility and stretchability [16]. A lower Young’s modulus of the originally rigid electrodes array can be realized through rational design of the electrode structure [17]. The requirements of safe and reliable long-term implantation will be achieved via the selection of the electrode and base material, and the design of the electrode structure. Figure 1 shows some typical neural electrodes. In order to better fit the nerve tissue, the nerve probe is required to be flexible, however, this flexible probe is difficult to safely and reliably implanted into the brain [18]. Implanting the electrode array into the brain requires not only mechanical performance with a certain degree of stiffness, but also minimally invasive surgery to achieve the smallest possible surgical coverage. It is also necessary to implant a large-flux and high-density electrode array in a short time. Not only that, but accurate and reliable implantation into the deep brain area has to be considered [19,20,21]. This study introduces the flexible method of neural microelectrodes, whether it is the choice of flexible materials or the design of special structures [22]. How to safely and reliably implant the flexible electrode is also enumerated. 

## 2. Flexible Materials for Neural Microelectrodes

### 2.1. Electrode Materials

**Figure 2:**
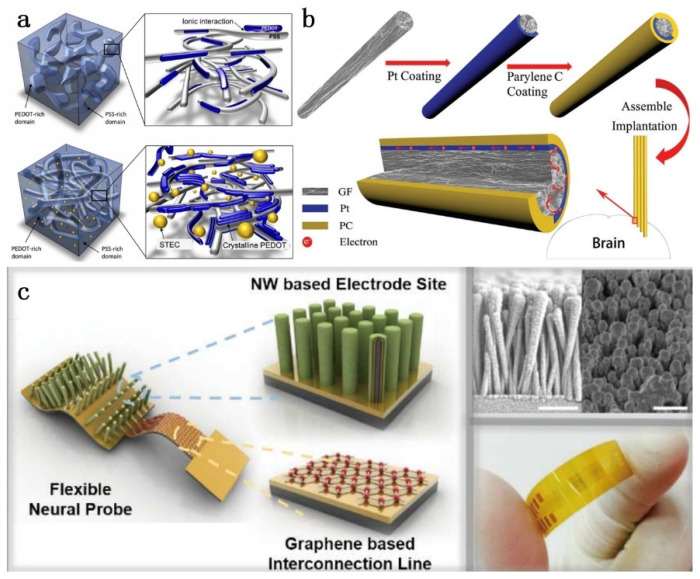
Electrode material: (**a**) PEDOT:PSS conductive polymer film (above without STEC, below with STEC [36]; (**b**) GF-Pt microelectrode [66]; and (**c**) nerve probes based on graphene, ZnO nanowires and conductive polymers [63].

The implantable flexible nerve microelectrode is mainly composed of a substrate, an electrode, and a package, each of which plays a crucial role in its performance. Due to its excellent chemical stability and high electrochemical performance, precious metals (gold, platinum, iridium, titanium, etc.) and their alloys are the most common electrodes [29]. The Young’s modulus of traditional solid material (metal) is usually several orders of magnitude higher than that of nerve tissue [14]. This mismatch between soft tissue and implant can cause the tissue to move slightly relative to the probe [30]. In addition, implants with a high Young’s modulus will cause tissue immune rejection, and the resulting glial scar will hinder the transmission of nerve signals [31]. Simultaneously, to minimize damage during implantation of the electrode, it is necessary to manufacture a considerably tiny electrode. However, the electrode with a smaller size contributes to an increase in impedance and a drop in charge storage capacity (CSC), which indicates poor-quality recording signal quality and a high stimulating current that will damage the tissue. In order to achieve long-term safety and reliable working, the electrode material should have the following properties: excellent electrical property [4] and low Young’s modulus [16,32,33]. Due to the inherent flexibility, stretchability, and high electrical performance, the conductive polymer is a good choice as an electrode material [16]. In addition, the mechanical properties of conductive polymers match those of biological tissues [15]. Conductive polymers as electrode materials also have a low impedance and high charge storage capacity [34,35]. For instance, PEDOT:PSS is an ideal electrode due to its high electrical conductivity, stretchability, and biocompatibility. Wang et al. [36] added conductivity-enhancing dopants in PEDOT:PSS to prepare a highly stretchable conductive polymer. Its electrical conductivity exceeds 3100 S·cm^−1^ at a strain of 0%, exceeding 4100 S·cm^−1^ at a strain of 100% and 100 S·cm^−1^ at a strain of 600%. Figure 2a shows a PEDOT:PSS conductive polymer film. The polythiophene (PT) copolymer has a maximum electrical conductivity of 10–100 S·cm^−1^ and a low Young’s modulus of 3 GPa [37], and the conductive polymer has an adjustable mechanical and electrical performance, which can adapt well to the biological environment and record neuron electrical signals for a long time [38]. 

Carbon-based materials mainly include glassy carbon, carbon fibers, carbon nanotubes, graphene, and laser-structured carbon, etc. This kind of material has a high electrical and mechanical performance. Since the interconnected porous channels inside the carbon are helpful to realize the rapid migration of electrons and ions, the electrochemical performance can be improved [39]. Graphene is a hexagonal 2D single-layer carbon atom sheet with an excellent electrical conductivity, biocompatibility, mechanical strength, optical transparency, and the advantages of inducing cell differentiation [40]. However, graphene has a relatively high Young’s modulus (about 1 TPa) [14]. The transfer and deposition of graphene from a rigid substrate to a flexible substrate is an effective method to obtain a flexible structure. Park et al. [41] thermally stacked graphene on a flexible Parylene-C film as an electrode, and the flexibility of the electrode was achieved. Castagnola et al. [42] introduced the glassy carbon (GC) microelectrode arrays on flexible polymer substrates through a carbon-MEMS (C-MEMS) microfabrication process followed by a novel pattern transfer. These implantable GC microelectrodes provide unique advantages in the electrochemical detection of dopamine (DA) and serotonin (5-HT). Yang et al. [43] introduced laser treated carbon nanotube yarn microelectrodes and obtained improved sensitivity of DA detection. Other electrode materials are summarized in Table 1 as follows. 

Shape memory alloys (SMAs) are functional materials with special properties. SMAs have the advantages of a high mechanical property, shape memory function, and biocompatibility [55]. As electrode materials, they can be well adapted to the biological environment [55]. Nickel titanium (NiTi), a biocompatible material with a shape memory effect and superelasticity, is the most widely used SMAs film, and has been applied in a variety of biomedical fields [55,56]. Zhao et al. [56] demonstrated a 3D expandable nickel-titanium alloy microwire electrode array, which can be designed into a desired shape to conform to the brain vascular structure and minimize vascular damage during implantation. Because the shape memory alloy of Nitinol has a shape memory effect and super elastic flexibility, it provides a new opportunity for the nerve interface without vascular damage in chronic animal research, and can achieve stable long-term recording of single spike signals and low-frequency pulse signals, while minimizing implant damage. Crampon et al. [57] introduced a new nerve cuff electrode with shape memory alloy (SMA) armature. SMA armature ensures the firm closing of the electrode, so the installation procedure is very simple. The SMA electrode can be adapted for safe close-fitting installation.

Liquid metal has a unique electrical conductivity and unparalleled adaptability to flexible systems [58,59]. Guo et al. [60] prepared a flexible neural microelectrode array based on gain alloy through embedding liquid metal electrodes and interconnecting wires into the PDMS membranes. The flexible nerve microelectrode array has a low Young’s modulus, low impedance, and biocompatibility similar to that of the nerve tissue. By implanting liquid metal electrodes into the peroneal nerve and tibial nerve of the bullfrog, and applying electrical stimulation, it was found that the hind limbs of the dead bullfrog are bent under electrical stimulation.

Simply studying flexible conductive materials is not the ultimate solution to all mechanical mismatch problems in biology, especially for implantable biological systems. Biomaterials exhibit highly different combinations of mechanical properties, for example, cortical bone (Young’s modulus ≈ 10 GPa) is hard and brittle, dura mater (Young’s modulus ≈ 1 MPa) is hard, and the spinal cord and brain tissue (Young’s modulus ≈ 100 Pa–10 kPa) are soft and flexible [61]. Ren et al. [61] developed a magnetically active fluid or slurry based on LM, which is formed by dispersing magnetic iron particles in a gallium-based LM matrix. The designed magnetic responsive LMMS material is designed to achieve multi-function in biological systems. Implanted electrodes provide a new mechanically adaptable bioelectrode system. Stiffness and viscosity of the electrode can be adjusted to match different mechanical properties of the tissue, significantly reducing the damage caused by the implanted electrode.

It is difficult for electrodes based on a single material to meet the complex biological environment. Organic electronic materials (such as conductive polymers) are essentially soft, stretchable, conductive or semi-conductive; however, compared with inorganic electronic (metal) materials, organic electronic materials have a lower electron mobility and conductivity. The mixing of different materials can achieve excellent conductivity and flexibility [16]. For example, silver nanoparticles are in-situ formed from silver flakes and are mixed in a polymer matrix. This method not only achieves soft and stretchable properties, but also increases the electrical conductivity of electronic devices by about 108 times [62]. Ryu et al. [63] demonstrated a neural probe based on graphene, zinc oxide nanowires, and conductive polymers. The hybrid structure of gold and graphene is used to achieve flexibility and high conductivity. The use of zinc oxide nanowires for increasing the effective surface area of electrode can significantly reduce the impedance value and improve the signal-to-noise ratio. The conductive polymer PEDOT coating improves the electrical characteristics of electrode, while providing higher biocompatibility. In vivo recordings of the nerve signals showed that the electrode array of the hybrid material could detect clearer signals. Lee et al. [64] successfully deposited PEDOT:PSS/GO composite materials onto gold microelectrodes. Compared with the PEDOT:PSS conductive polymer, the composite material has better electrical properties and flexibility, while being more mechanically durable. This kind of mixing and integrating materials with different functions on the neuro microelectrode can not only achieve good electrical conductivity, but also meets the soft and stretchable properties. Table 2 summarizes the influence of doping electrode materials on electrode performance.

### 2.2. Substrate Material

Substrate materials with specific properties such as flexibility, biocompatibility, and stability are expected for the chronic implantation of electrodes in the complex environment of the body [15]. For all neural microelectrodes, the biocompatibility of substrate materials is a prerequisite, not only for the microelectrode’s long-term stability, but also for safety. An ideal substrate material should be non-cytotoxic in vivo and release no substances. Evaluation of the material’s biocompatibility includes the test of cytotoxicity, acute/chronic systemic toxicity, sensitization, and hemocompatibility, etc. The implant-induced inflammatory response has a certain effect on the performance and lifetime of implanted microelectrode. In addition, Young’s modulus of the implantable neural microelectrode is extremely important for in vivo applications [11]. The introduction part explains that the mismatch of Young’s modulus between soft tissue and electrode will trigger immune reactions, resulting in the electrode not being able to achieve long-term safety and reliable working. The material’s stability is another important consideration of implantable neural microelectrodes. The fabrication imperfection of the electrode and substrate, such as unavoidable pinholes and defects, will cause oxidation and delamination of the materials, which could shorten the longevity of the microelectrode in vivo environments [15]. 

**Figure 3:**
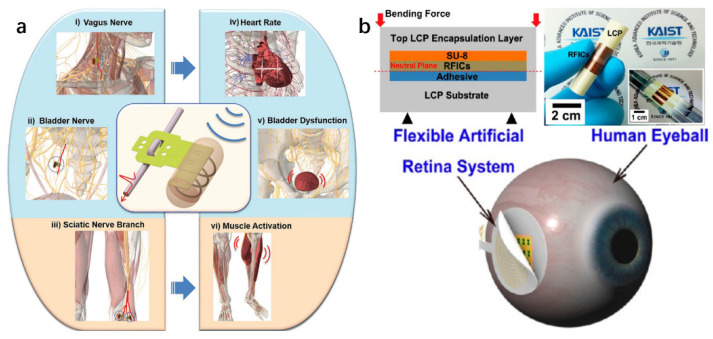
Flexible neural electrode based on polymer substrate material: (**a**) flexible nerve clip (FNC) electrode based on polyimide [76], and (**b**) flexible neural electrode based on LCP [90].

Polymer is the most widely used flexible substrate material. The material generally has the properties of flexibility, excellent biocompatibility, and insulation, and is not easy to break [70,71]. Common polymer materials include polyimide (PI), polydimethylsiloxane (PDMS), parylene, SU-8, liquid crystal polymer (LCP), cellulose nanocomposites, etc. [72]. PDMS is the most widely used substrate material [73] and offers high biocompatibility, excellent insulation, and high conformability with the tissue. It also has the advantage of being low cost. In addition, PDMS is one of few materials tested for long-term implantation [74]. Adly et al. [75] introduced the approach of printing high-resolution carbon microelectrode arrays (MEAs) on soft substrates, including PDMS and hydrogel. MEAs were applied for localized recordings of the action potentials from HL-1 cells. PI has a higher tensile strength of 390 MPa, low Young’s modulus of 8.37 GPa, and considerably high biocompatibility [15]. Lee et al. [76] used polyimide as the base material to prepare a new type of flexible nerve clip electrode (FNC), which can be connected to a variety of peripheral nerve fibers. Compared with the traditional cuff electrode, the flexible nerve clip electrode can stimulate the pelvic nerve, vagus nerve, and sciatic nerve, and also has a regulatory effect on some physiological activities. This polymer-based nerve electrode can be conformally attached to the brain tissue without damaging nerve cells. Figure 3a shows the flexible nerve clip (FNC) electrode based on polyimide. In addition, some new materials that are more compliant have appeared. Liu et al. [77] used perfluoropolyether dimethacrylate (PFPE-DMA) as the packaging material of IFNEs, and the Young’s modulus of PFPE-DMA is two to six orders of magnitude lower than those of PI and PDMS. Zhang et al. [78] developed a 3D twining electrode using intelligent shape memory polymers (SMPs), which has permanent shape reconfigurability (from 2D to 3D), distinct controllability of Young’s modulus (from ~100 MPa to ~300 kPa), and shape memory recoverability at body temperature. The proposed 3D twining electrodes can dramatically reduce the nerve injury associated with the Young’s modulus and geometrical mismatches. Ware et al. [79] introduced a method using photolithography to pattern thin-film flexible electronics on shape memory polymer substrates, and obtained improved biocompatibility and flexibility of the Au electrode. However, all pure polymer substrate materials have their own shortcomings, which seriously affect the normal work of neural microelectrodes in the body. For example, the surface of the polyimide film has poor hydrophilicity and durability, which will cause the adhesion of the polyimide to the metal electrode to decrease [80]. Parylene has poor adhesion to most materials due to its inertness, which impairs its long-term stability in the biological environment [81]. PDMS as a base material also has certain problems. Because its thermal expansion coefficient is very different from that of metal, when high energy is applied to PDMS during the metal patterning process (such as sputtering, and ultraviolet exposure), metal pattern microcracks are prone to appear, and the electrical connection of the metal pattern with cracks has a lower stability and higher resistance than the pattern without cracks [82]. Therefore, modifying the polymer substrate material can improve these problems. In order to overcome the problems in the manufacture of PDMS electrodes, Chou et al. [82] suggested the use of a parylene-C intermediate layer, which prevents cracks in the metal layer, so that metal patterns can be manufactured on PDMS more reproducibly and reliably. The modification of the base material can also improve the flexibility of the material and reduce the Young’s modulus. A summary of the relevant polymeric substrates is shown in Table 3.

## 3. Structure Design of Nerve Microelectrode

As described above, selecting electrode materials and base materials with a low Young’s modulus can significantly reduce the rigidity of the nerve electrode probe. However, the design of the electrode structure is also important because the structure determines the interaction properties of the neural interface. For example, through proper structural design, a material with a higher Young’s modulus can exhibit better flexibility than a material with a lower Young’s modulus. At the same time, the design of the electrode structure can reduce the damage of the tissue. The electrical signals of the brain are obtained using various electrodes. Table 4 shows the different types of extracellular electrical signal of the brain. Below, we will introduce the structure design of the nerve electrode [91].

### 3.1. Linear Banded Structure

**Figure 4:**
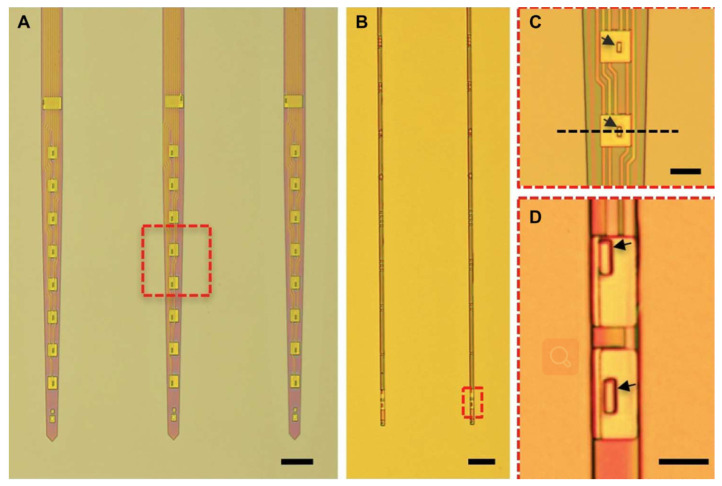
Nanoelectronic thread (NET) electrodes [18]: (**A**) as-fabricated NET-50 probes on substrates, (**B**) as-fabricated NET-10 probes on substrates, (**C**) zoom-in views of electrode as marked by the dashed boxes in (**A**), and (**D**) zoom-in views of electrode as marked by the dashed boxes in (**B**).

The initial implantation injury to the neural tissue is directly related to the cross-sectional footprint of the probes. The mean spacing of the blood microvessels in the rat brain is shown to be ≈50 μm, and the mean distance between neuron and the closest microvessel is ≈15 μm [13,93]. The reduction of the cross-sectional area of the implanted electrode can reduce the damage of the blood vessels and the local neuronal environment. Kipke and co-workers [94] developed an ultra-small, implantable carbon fiber microelectrode with a cross-sectional diameter of only 8.5 μm. The carbon fiber microelectrode consists of carbon fiber core coated with poly (p-xylylene) (parylene N) as the dielectric insulation layer and poly (ethylene glycol) methacrylate as the anti-biofouling layer. The results indicate that the Young’s modulus of the carbon fiber microelectrode is lower than that of a conventional silicon probe. Due to its ultra-small cross-sectional footprint and lower Young’s modulus, the carbon fiber microelectrode caused limited damage to the blood vessels after implantation and dramatically reduced reactive tissue responses two weeks post-implantation as compared with conventional silicon probes. Zhao et al. [56] developed a 3D expandable nitinol microwire electrode array that can be designed to the shape of conforming to the brain vasculature, which can reduce the damage of the blood vessels during implantation. Luan et al. [18] developed a SU-8 based polymer substrate with a cross-sectional area of 10 × 1.5 μm. The smaller cross-sectional area of the probe can further weaken the damage of the tissue. Their research shows that the electrode can reliably detect and track a single cell for several months, while the impedance, noise level, and signal recording quality of the electrode remain stable. Figure 4 shows the nanoelectronic thread (NET) electrodes.

**Figure 5:**
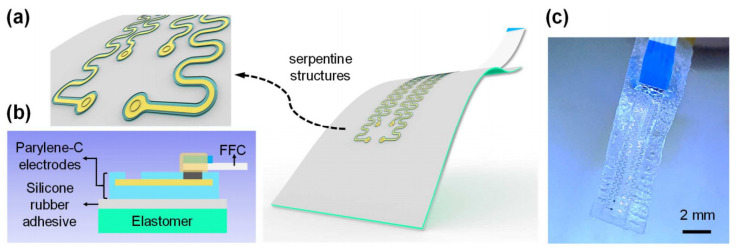
Design of the stretchable electrodes [100]: (**a**) schematic illustration of the stretchable electrodes with serpentine structures; (**b**) geometric layout of the stretchable electrodes; (**c**) picture of the assembled device.

In addition, the appearance of the wavy structure, serpentine structure, and island bridge structure of the electrode also achieves the flexible and stretchable properties of the nerve probe [95]. By bonding the inorganic film to a pre-strained elastic substrate, an electrode array with a wavy structure with extremely stretchable and flexible properties can be obtained. Qi et al. [96] designed a wave-shaped stretchable electrode. This unique wavy structure uses a pre-stretched PDMS tripod structure as the substrate and transfers the gold nanoribbons to the surface of the PDMS tripod. This structural design can significantly reduce the stress concentration on the gold electrode. This wavy structure can realize highly conductive, stretchable, and flowable electrodes. Connect this flexible electrode to the curved surface of the rat to record intracranial electroencephalogram or electrocorticogram signals. However, due to the change in band gap of the film caused by the strain at the peaks and troughs of the wavy structure, the wavy structure cannot obtain great stretchability [34,97]. Due to the high tensile property, the serpentine design is the most widely investigated structure for stretchable electronic products [98]. The serpentine interconnection is composed of multiple periodically distributed units. The unit includes two semicircles interconnected by straight lines. The semicircles can rotate in a plane or bend out of a plane to reduce the strain of the serpentine material at tensile strain [99]. One problem with this serpentine structure is the bond strength between the serpentine interconnection and the elastic substrate. Ji et al. [100] successfully transferred the printed serpentine parylene-C electrode to any elastic substrate by introducing a thin layer of low modulus silicone rubber adhesive. The smallest change in electrochemical impedance of the microelectrode after 5000 times of repeated loading proves its reliability. Figure 5 shows the stretchable electrodes.

### 3.2. Ultra Thin Electrode

**Figure 6:**
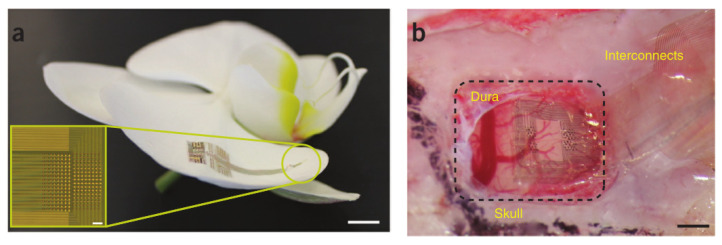
NeuroGrid structure and spike recordings in freely moving rats [104]: (**a**) The NeuroGrid conforms to the surface of an orchid petal (scale bar, 5 mm). Inset, optical micrograph of a 256-electrode NeuroGrid (scale bar, 100 µm). Electrodes are 10 × 10 µm^2^ with 30-µm interelectrode spacing. (**b**) The NeuroGrid conforms to the surface of the rat somatosensory cortex. The edge of the resected dura is visible at top left of the craniotomy (scale bar, 1 mm).

The method of reducing the thickness has been successfully used to fabricate flexible neural microelectrodes [17]. For some rigid materials, the flexibility can be achieved by reducing the thickness of the material to prepare the ultra-thin plane. These ultra-thin planar electrodes can be attached to the curved surface of the nerve tissue for long-term recording of ECoG, LFP [101]. Muller et al. [102] fabricated a thin-film, high-density multi-electrode array to record ECoG from the human cortical surface. High-density electrocorticography (ECoG) arrays are promising devices for high-resolution neural recording from the cortical surface. Khodagholy et al. [103] prepared ultra-thin 2D planar electrode array based on a poly (p-xylene) polymer substrate, and the thickness of the electrode was only 4 μm. Because of its thin thickness, the electrode is more flexible and can carry out ECoG on the somatosensory cortex of rats for a long time after implantation. The NeuroGrid electrode has the advantages of high biocompatibility, super integration, expandable channel number, and ultra-high spatial resolution. Khodagholy et al. [104] prepared an ultra-thin neural grid electrode. NeuroGrid contains 256 electrodes with an area of 10 μm × 10 μm (to match the size of neuron), a spacing of 30 μm, and an electrode thickness of 4 μm, as shown in Figure 6. Their research shows that neural grid electrodes can record LFP for a long time. This ultra-thin electrode exhibits excellent mechanical compliance and structural durability at deformation [17].

### 3.3. Mesh Structure

The effective connection between the nerve electrode and the nerve tissue depends on the close and seamless contact between the two [13]. However, the appearance, structure, or shape of the electrode probe we prepared is not similar as brain tissue, which makes it difficult for the electrode probe to achieve conformal contact with the brain tissue. In order to bridge the difference between nerve tissue and electronic devices, the electrode array is designed into a mesh structure, which can behave more like nerve tissue [105]. For example, the mesh electrode has a more flexible nature, has a three-dimensional (3D) structure (the structure of nerve tissue is usually 3D [106]), and less damage to nerve cells.

**Figure 7:**
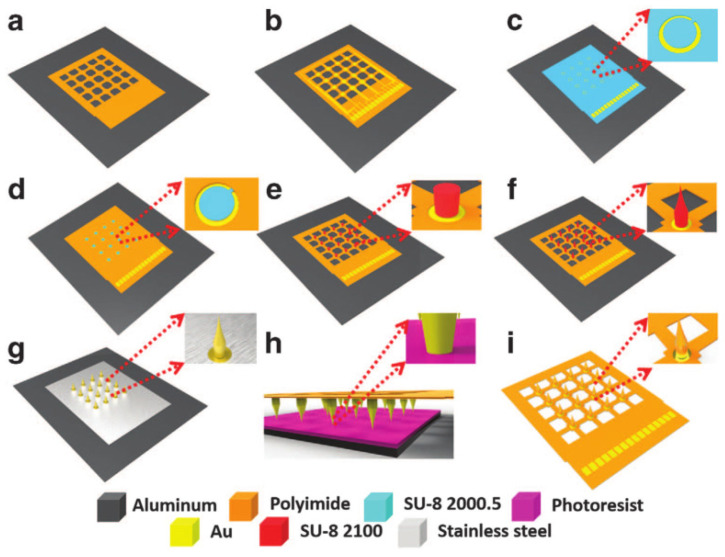
Fabrication process for the flexible microneedle electrode [106]: (**a**) bottom layer defined by UV lithography technology; (**b**) metal tracing formed by lift-off process; (**c**) SU-8 adhesion layer patterning; (**d**) top layer defined by UV lithography technology; (**e**) SU-8 pillar array formed by UV lithography technology; (**f**) SU-8 sharp tips formed by drawing lithography technology; (**g**) gold layer deposition on the surface of microneedle electrode; (**h**) parylene insulation layer deposition on the microneedle electrode; (**i**) electrode release from the substrate.

Xiang et al. [106] developed an electrode array with a 3D network structure, as shown in Figure 7. The electrode array is prepared on a flexible mesh substrate through simple micro-machining technology. There are some microneedle tip electrode probes on the flat mesh substrate, and these microneedle tip electrodes are manufactured by photolithography technology. Because the electrode probe has sufficient rigidity, the microneedle can penetrate the nerve tissue and record nerve signals from different functional layers. The mesh substrate has good flexibility, so that the electrode array is in conformal and seamless contact with the brain plane. Experiments on rats have proven that the mesh electrode array can successfully contact the curved brain plane of rats and record the LFP and spike signals of rats. In order to prepare a more flexible 3D electrode array, the holes of the mesh electrode can be enlarged. Meanwhile, 3D macroporous electronic device arrays can function as scaffolds and allow for 3D interpenetration of cultured neuron cell networks without an adverse effect on cell viability [107], which can show better biocompatibility. Xie and co-workers [108] studied the 3D microporous nanoelectronic networks. Their fabrication exploits conventional planar 2D lithography with a sacrificial layer etched to yield free-standing microporous nanoelectronic probe. The mesh design is unique in having a two-dimensional (2D) open area of ~80%, feature sizes to sub-10 µm scale, and importantly, a high flexibility with an effective bending stiffness of <0.64 × 10^−15^ N m^2^, four to seven orders of magnitude smaller than conventional carbon fiber, Si, and thin polyimide neural probes.

### 3.4. Origami and Kirigami

**Figure 8:**
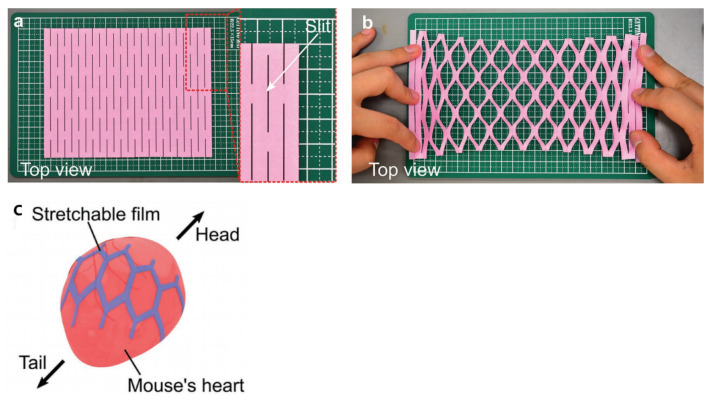
“Kirigami” design-based parylene film [111]: (**a**,**b**) Photographs and schematics of stretching of a kirigami paper (120 mm × 160 mm) with a slit pattern formed by scissors ((**a**): before and (**b**): after stretching). Schematic of “before stretching” (**a**) includes a cell unit (purple colored), which is used in the modeling. (**c**) Schematic and photographs of a fabricated parylene film placed over a beating mouse heart.

In addition to the above-mentioned electrode structure, the shape-adaptability of the structure formed by origami/kirigami provides an interesting method for scalable/flexible electronics [109]. “Origami” (ori means “folding” and gami means “paper”) [110] and “Kirigami” (kiri means “cutting”) are artistic transformations from a flat film/sheet into numerous 2D and 3D sculptures by folding, cutting, and gluing techniques. Yusuke Morikawa et al. [111] found, in addition to a super-stretched film structure, the electrode can follow the changes of biological tissues (such as brain and heart tissue) and deform, as shown in Figure 8. The electrode is made of parylene polymer with a good biocompatibility as the base material, and non-stretchable metal material Pt/Ti as the electrode material. However, the film showed a highly stretchable property by patterning the slit into a kirigami design. A Pt/Ti microelectrode array embedded in 11 µm thick parylene film with 5 × 91 slits exhibits a film strain of ≈250% at 9 mN strain-force (0.08 MPa in stress) with a Young’s modulus of 23 kPa, while the 3 × 91-slit film has a Young’s modulus of 3.6 kPa. The maximum strains of these devices are ≈470% and ≈840%, respectively. The electrode of this structure is attached to the heart of the mouse, which can record the ECoG in the body very well. Origami technology can also produce special geometric structures and enhanced sensing corresponding electrical sensors [112]. Yan et al. [113] used origami technology to prepare a 3D cage structure of neural microelectrodes (with SU-8 as the base material). The electrode probe is integrated on the outer surface of each leg of the 3D cage. The electrode array can explore their electrophysiological activities by electrically stimulating DRG cells and then recording their action potential responses in real time. Goshi et al. [114] reported a technology for microfabricating 3D origami-styled micro electro mechanical systems (MEMS) structures with glassy carbon (GC) features and a supporting polyimide substrate. Kirigami and origami art not only have a wide range of applications on a macro scale, but their application on the micro scale significantly improves the stretchability and flexibility of the nerve electrode.

### 3.5. 2D/3D Electrode Structure

2D planar electrodes and 3D penetrating electrodes are the most common implantable neural microelectrodes. 2D planar electrodes are mainly attached to the plane of the brain to record ECoG electrical signals. They are less invasive and can achieve long-term electrical signal recording, but the disadvantage is that the spatial resolution of the electrode array is low [115]. The 3D penetrating electrode array can record a single unit motion signal with a high spatial and temporal resolution. Spanu et al. [116] developed a 3D microelectrode array (3D-MEA) specifically designed for brain-on-a-dish applications. The 3D-MEA consists of pillar-shaped gold microelectrodes realized by electroplating directly on top of a standard MEA. The 3D-MEA structure successfully recorded both epileptiform-like discharges (upon the administration of 4-AP) and electrically-evoked neuronal activity. However, it cannot detect such a large number of neuronal cells, because a large number of implanted electrode probes will cause damage to the brain tissue [117]. To better understand how the brain converts neuronal signals into actions, behaviors, and motivations, it is necessary to record neuronal electrophysiological signals from multiple areas of the brain [118]. Therefore, it is a good solution to integrate 2D planar electrodes and 3D penetrating electrode arrays. 

Goshi et al. [114] introduced a neural signal recording microelectrode array that integrates surface (cortical) and deep (intracortical) GC microelectrodes into a single flexible thin film device. The electrode array was originally prepared with a 2D geometric structure. When the device is unfolded, the pre-formed polyimide handle will automatically separate from the flat substrate and form the penetration part of the electrode in a 3D manner. This kind of electrode array with flat and penetrating parts can record the electrical signals of neurons from the brain surface (ECoG) and cortex (single unit action potential) well. By recording typical somatosensory evoked potentials (beard deflection) in rats, it is preliminarily confirmed that the 2D and 3D electrodes have similar trends in the signal waveforms of spontaneous and stimulated activities. Vahidi and co-workers [119] also introduced an in vitro-in vivo neural probe, which provides a compelling platform for study of neural coding and stimulation coding/reconstruction. The probe uses 2D thin film micro-manufacturing technology to combine the outer (surface) and inner (depth) microelectrode arrays (in the cortex) of the cortex, and expand into an origami 3D-like probe during brain implantation. Epi-Intra (outside-inside) probes record broadband activity from the surface and deep parts of the brain, including single unit activity (SUA), local field potentials (LFPs) signals and multiple unit activity (MUA). This probe records CRF, which is an excellent candidate for neural coding and understanding the relationship between sensory neuron responses and their stimuli (stimulus codes).

The structural design of electrode flexibility is introduced above, for example through a linear structure and ribbon structure with reduced cross-sectional area, flat membrane electrode with ultra-thin thickness, mesh electrode structure with microporous structure, and flexible stretchable structure designed by paper cutting and origami. The design of these electrode structures can not only reduce the rigidity of the electrode, but also achieve stretchable properties. At the same time, it is also very helpful for reducing tissue damage. Finally, the 2D/3D electrode structure is introduced. The electrode of this 2D/3D electrode structure has the ability to record multiple functional areas of the brain.

## 4. Implantation of Flexible Neural Microelectrode

The traditional rigid nerve microelectrode will damage nerve tissue cells during implantation. At the same time, the mismatch of Young’s modulus between the brain and the implanted electrode can induce an inflammatory reaction and wrap a layer of glial scar around the electrode. This kind of colloidal scar will lead to the insulation and failure of electrode equipment. Studies have shown that flexible implantable neural microelectrodes can limit severe foreign body reactions [120,121]. In the first half of this paper, how to make an implantable neural microelectrode flexible is described. However, the soft nerve probe cannot meet the mechanical stiffness of implantation. To successfully implant the flexible nerve probe into brain tissue, the probe must have enough mechanical strength. 

From an ideal mechanical point of view, neural probes will be implanted if the maximum compression force that can withstand before bending (called buckling force) is higher than the minimum force required for insertion in the soft tissue (called the insertion force) [22]. The buckling force of the neural probes can be calculated theoretically using Euler’s formula:

(1) $$F_{buckling}=\frac{\pi^{2}EI_{x}}{{\left(KL\right)}^{2}}$$

In the formula, *F_bucklingis_* is the buckling force, *E* is the Young’s modulus of the material, *I_x_* is the moment of inertia of surface contact, *K* is the effective length coefficient, and *L* is the unsupported length of the beam of the probes. Consequently, to successfully implant the flexible nerve probe into brain tissue, one or several strategies can be chosen: the *E* value of probe insertion can be improved by temporary coating or removable auxiliary equipment [85]; the critical buckling force of probe can be increased using microfluidic drive during implantation process; the stiffness of probe can be enhanced by choosing stimulus responsive materials as the encapsulation layer of the probe; and the *L* value can be significantly reduced through a guiding device, which increases the bucking force. 

### 4.1. Temporary Coating

The most common method to enhance the stiffness of flexible probes is by coating bioabsorbable polymer. During implantation, the biodegradable material acts as a temporary coating to provide temporary stiffness to prevent the probe from bending. When the probe is implanted, the physiological fluid will degrade and absorb the polymer material [22]. PEG (polyethylene glycol) is used as an insertion aid because of its good biocompatibility and its ability to be degraded and absorbed in the tissue fluid [122,123]. However, the rigidity of PEG is limited. To meet the rigidity during implantation, the thickness of the coating needs to be increased. However, this method will amplify the tissue damage during implantation. At the same time, PLGA is also biodegradable and biocompatible, and is often used as a reinforcing agent to temporarily reinforce the implantable flexible probe [71]. However, the degradation time of PLGA is generally 3 to 4 weeks, which exceeds the time for the occurrence of polar tissue reactions or even chronic tissue reactions. Lo et al. [124] developed a micromolding method for coating a non-functional miniaturized SU-8 probe with an ultrafast degrading tyrosine-derived polycarbonate (E5005(2K)). The coating can provide sufficient rigidity for the insertion of the device, and at the same time quickly degrade (within a few hours) to record nerve signals for a long time. However, coating materials with a faster degradation time will result in only one chance of implantation. In order to better meet the requirements of flexible probe implantation. Biodegradable polymers with an adjustable degradation rate and mechanical stiffness have attracted more attention. Kil et al. [125] achieved the purpose of adjusting the dissolution rate and mechanical hardness by controlling the chain length of the dextran molecular chain. Their research showed that using dextran as a coating material enhances the mechanical strength of the flexible probe and prevents the probe from bending. Four months after implantation, very limited glial scar tissue was formed, and the density of neuronal tissue at the implantation site did not decrease significantly. This confirms the utility of dextran as a biodegradable material to temporarily increase strength. The PLGA described above as a temporary coating has the disadvantage of a slower degradation time, but it can be combined with a polymer material with a faster degradation rate, so that a suitable degradation time can be achieved. Jolien et al. [126] provided an implantation method with a double-layer biodegradable polymer material. This implantation method uses two different bioabsorbable polymer materials. These two materials are PVA and PLGA, which can facilitate the implantation of microelectrode probes. The rigid PVA material provides the hardness required to penetrate the brain, and the temporary coating of the double-layer structure has a suitable degradation time so that it can be inserted a few millimeters deep into the pig’s brain.

**Figure 9:**
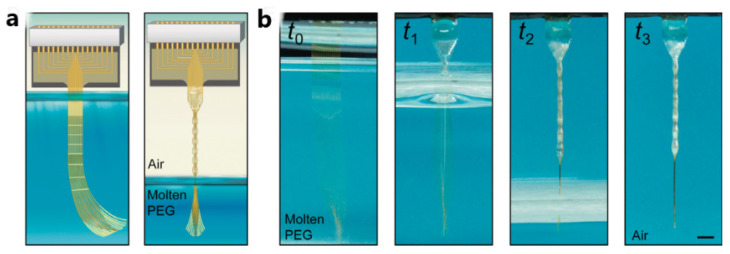
Elastocapillary self-assembly and PEG temporary coating of Neurotassels [132]: (**a**) schematics of the elastocapillary self-assembly of a Neurotassel, and (**b**) time sequence photographs of the elastocapillary self-assembly of a Neurotassel. Scale bar, 1 mm.

The combination of the degradable and absorbent polymer material and the elastic capillary interaction can realize the implantation of the flexible electrode well. Elastic capillary self-assembly is an efficient and expandable process that arranges high-aspect-ratio and flexible building blocks into an ordered structure through long-range capillary action [127,128], which is widely observed in natural systems (such as wet hair) and engineering systems from micropillars to carbon nanotubes [129,130,131]. Guan et al. [132] developed a nerve tassel composed of a series of high-aspect-ratio flexible microelectrode wires. When these microelectrode wires are taken out of the degradable and absorbable polymer, nerve tassels can spontaneously assemble into thin implantable fibers by elastic capillary interaction. Due to the sclerosing effect of PEG, nerve tassels/PEG fibers can be implanted into the brain area of mice. After implantation, PEG will dissolve in the brain and body fluid, and the nerve tassel electrode will be transformed into a highly flexible microelectrode wire. The long-term implanted nerve tassel electrode causes minimal neuronal cell damage in the brain, and can stably record the neural activity signals of the mice that are learning to perform tasks. Figure 9 shows the elastocapillary self-assembly and PEG temporary coating of Neurotassels.

As mentioned above, the use of degradable and absorbent polymer materials can temporarily increase the rigidity of the implanted probe and facilitate the implantation of the flexible probe. In particular, a coating material with an adjustable degradation rate and mechanical stiffness is used. This material can adjust the degradation rate and mechanical stiffness of polymer according to the requirements of implantation depth to meet the requirements of implantation. However, this coating material will also significantly increase the cross-sectional area of the implanted probe and the serious damage caused by the insertion [133]. 

### 4.2. Removable Auxiliary Equipment

The use of removable shuttles is also a common implantation method. The use of a movable shuttle increases the cross-sectional area of the implanted probe, but allows for the use of the hardest materials. Therefore, it can be the theoretical minimum size when inserting any flexible device. Therefore, using a rigid shuttle for insertion is the most attractive option in inserting flexible devices currently [134]. Removable shuttles are composed of two parts: one is the shuttle material with appropriate rigidity, and the other is to adhere the flexible electrode to the shuttle. The rigid material inserted into the shuttle is usually silicon [135], stainless steel [18], or tungsten wire [136]. A material with greater rigidity can achieve a smaller cross-sectional area. Coupling methods include polyethylene glycol coupling [18], electrostatic force [137], or direct physical coupling [138].

**Figure 10:**
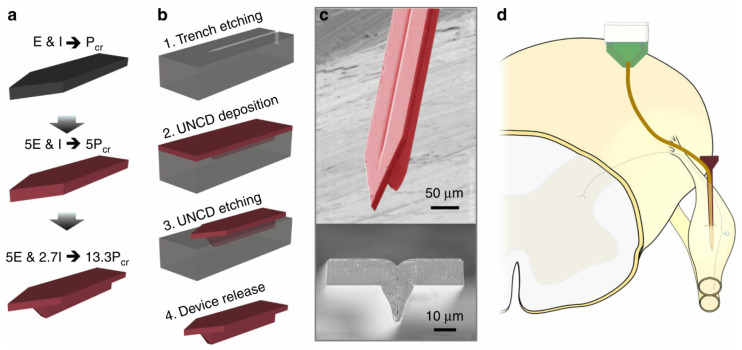
Ultra-nano-crystalline diamond (UNCD) shuttle [142]: (**a**) progression of design improvement from a simple silicon shuttle, with a buckling load of *P_cr_*, to an improvement of 13.3*P_cr_ by changing the Young’s Modulus, *E*, and moment of inertia (I); (**b**) process flow of a UNCD shuttle requiring only two masks (not shown) at steps 1 and 3; (**c**) SEM of released UNCD shuttle; and (**d**) insertion of a flexible electrode array into a dorsal root ganglia (DRG) using a rigid shuttle and retraction.

Felix et al. [135] described a method of temporarily fixing a silicon reinforcement with biosoluble PEG. The silicon shuttle can be released from the probe shortly after the probe is implanted. In order to better realize the implantation of flexible probes, thicker auxiliary implantation equipment will have a higher critical load and will be easier to penetrate the meninges for implantation. However, this is not advisable, because it will compress a larger area of brain tissue during insertion and destroy more of the vascular system [139]. Making a sharper tip without increasing the cross-sectional area of the insertion device is a good way to promote implantation [140]. Joo et al. [141] described a novel design and manufacturing process to manufacture a 3D sharp silicon shuttle. This 3D tip silicon shuttle machine can penetrate the dura mater of rats, allowing for faster, easier, and less damaging implantation of the flexible probes. The patterned photoresist is reflowed, and then its oblique profile is transferred to silicon by dry etching in order to obtain a sharp profile. This device reduces the implantation time and the risk of blood-brain barrier damage. When solving problems such as minimizing cross-sectional area and tissue compression, in addition to changing the tip shape of the silicon shuttle, you can also choose a harder material. Diamond is much harder than silicon-based materials. Na et al. [142] built a 3D diamond shuttle machine that can deliver super-compliant polymer microelectrodes (4.5 μm thick) through the dura and thick epineurium, as shown in Figure 10. Compared with the silicon bobbin of the same stiffness, the cross-sectional area of the diamond bobbin is 54% less. The results of their simulation reduced blood vessel damage by 37%. They also found that regardless of the speed of insertion, they could significantly reduce tissue compression.

Most neural probes have a limited stem length, preventing them from reaching many deep structures of the brain. The main reason for this is that the extra-long stem may bend or break during the implantation process. In order to implant the nerve probe into a deeper part of the brain, Zhao et al. [143] reported a deep nerve probe with an ultra-long penetrating handle based on a novel and simple parylene tubular structure. The handle body is made of a hollow parylene tube. During implantation process, a wire can be inserted into the tube to reinforce the handle and increase its strength for insertion into the nerve tissue. Tip of the tube can be closed or open. When the tip is opened, the extended metal needle can promote piercing of the hard brain tissue. The metal wire can be removed after implantation, leaving only the parylene tube in the brain tissue. Flexibility of the parylene handle will improve the chronic stable response. Implanting it into the amygdala of rats allowed for recording the nerve signals very well. Zhou and co-workers [144] developed an implantation method like a syringe. The flexible mesh electrode is put into the injection tube, and then the injection tube provides rigidity during implantation. After the injection tube is inserted into the brain, the flexible mesh electrode array is pushed out, and finally the injection tube is pulled out. Their research shows that the implanted mesh electrode array will cause very little inflammation and damage to the peripheral neurons in a short period of time.

Connecting a soft probe to a hard shuttle can help the probe to be implanted in a specific location, whether it is on the surface of the brain or in the deep brain. However, the final recording position of the probe after implantation will cause deviation of the recording position due to the retraction of the shuttle, which will affect the signal recording of the electrode probe [145]. Moreover, the increased rigidity and penetration area of the reinforcement-electrode assembly will aggravate acute and chronic injuries, destroy nearby neurons, and destroy blood-brain barrier [146].

### 4.3. Microfluidic Drive

**Figure 11:**
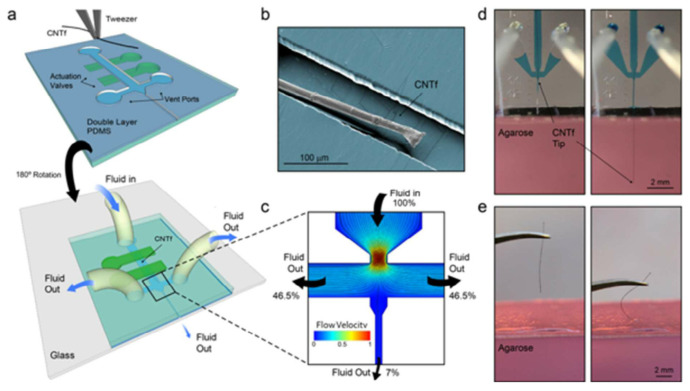
Device layout and microfluidic-assisted insertion of flexible CNTf microelectrodes in vitro [148]: (**a**) Schematic of the two-layer PDMS microfluidic device. Microelectrodes are placed and aligned manually inside the channel (top). The device is then inverted and bonded to a glass substrate (bottom). Push-down actuation valves (green) provide on-chip flow control. (**b**) False-colored SEM image of a 12 μm diameter microelectrode inside the PDMS channel. (**c**) Velocity field and flow pathlines in the microfluidic device. More than 93% of the total volume of fluid injected is deviated to the side venting ports, which minimizes the amount of fluid delivered to the outlet channel. (**d**) Microfluidic-assisted insertion of a 12-μm microelectrode in a brain phantom: the drag force produced by the fluid drives the fiber 4.5 mm into the phantom, without evidence of bending. (**e**) When mechanical insertion is attempted, the microelectrode irreversibly buckles upon contact with the agarose surface and does not penetrate inside the phantom.

The use of flexible probe insertion aids increases tissue damage and limits the advantages of flexible nerve microelectrodes [138]. The use of a microfluidic drive device can provide the necessary force for the flexible probe to penetrate the pia mater through the fluid force. This implantation method can achieve implantation without using implantation aids [147]. Vitale and co-workers [148] specially designed a microfluidic driven implant device, as shown in Figure 11. This device can apply tension to the flexible electrode array to prevent the electrode from bending, without increasing the cross-sectional area and rigidity of the electrode during the implantation process. In addition, this type of microfluidic drive device enables us to accurately implant electrodes with micron-level precision. They used copy molding technology to make a microfluidic drive device from two layers of PDMS. It works as follows: First place the carbon nanotube fiber (CNTf) microelectrode manually in the center of the fluid channel, and then glue the channel to glass substrate. The liquid flowing in the microfluidic channel exerts a viscous drag force on microelectrode due to the difference in speed, which keeps the CNTf under tension. The finite element simulation shows that distribution of the distributed load is no longer uniform, but increases linearly or quadratically along the length of probe. It is expected that the critical buckling force of the probe will increase by 16–30.59 times. The electrode probe of this implantation method can be implanted into the rat brain to a depth of more than 4 mm, and spontaneous individual unit activity can be detected in the cortex and subcortical area. Compared with syringe injection, the microfluidic drive device does not penetrate the brain and prevents changes in intracranial pressure by shunting fluid from the implant site during insertion and drive. The above-mentioned implantation device provides the necessary rigidity for the flexible probe to be implanted into the brain tissue through fluid force. The preparation of the embedded microfluidic channel can dynamically adjust the stiffness of the nerve probe by controlling the fluid pressure. Rezaei et al. [149] developed an embedded microchannel fluid implantation technology. The use of embedded microchannel fluid technology can dynamically control the stiffness of the probe by adjusting the polymer material, the size of the nerve probe, and the pressure of the fluid in the microchannel, so that they can be easily implanted into the target tissue. After implantation, the flexibility is restored by changing the pressure of the fluid.

The microfluidic device can implant flexible electrodes without using external supports and hardeners, avoiding additional tissue damage. However, it is still difficult to use this device to perform micro-positioning, although micrometer-level precise implantation has been achieved. Moreover, the feasibility of implanting longer electrodes is unclear. In addition, it is very difficult to make contact with microelectrodes after implantation, and during this connection, nerve signals are often lost due to micromotion [150].

### 4.4. Stimulus Responsive Materials

The development of smart materials has provided great help for the implantation of neural microelectrodes. Smart polymer materials can adjust their mechanical stiffness according to different stimuli (light, temperature, humidity, etc.) [151,152]. Wen et al. [153] prepared a neural probe with a variable Young’s modulus by using liquid metal, Ga, and an elastic substrate. The probe can be safely implanted into the brain area without external auxiliary implant tools or a sclerosing agent. This variable Young’s modulus utilizes the solid-liquid phase transition of the metal at body temperature and the shape modification of the probe to obtain an adjustable stiffness of five orders of magnitude. Under the condition of cooling, the probe is implanted into the brain of a rat with a depth of 2 cm. When Ga melts at physiological temperature, the probe becomes super soft and can adapt well to the Young’s modulus of brain tissue.

Some smart materials can also adjust their own Young’s modulus based on the response of water. Tang et al. [154] created a new type of microfibrous nerve probe (MFNP) with a variable Young’s modulus before and after implantation. The electrode probe has a coaxial structure, with CNTfs as the core electrode, and calcium ion cross-linked sodium alginate (SA) as the outer shell. The Young’s modulus of dry MFNP is 9.5 ± 0.5 GPa, 10^6^ times that of brain tissue (3.0 ± 0.3 kPa). Therefore, it can be implanted without any external auxiliary equipment or hardener. After implantation, the dry MFNP becomes soft after absorbing water, and its Young’s modulus is as small as 7.9 ± 3.1 kPa, which is close to the brain tissue and is called wet MFNP. The wet MFNP can move synchronously with the brain tissue, thus providing a stable interface, and finally a highly stable neuron signal reading, especially in long-term applications.

### 4.5. Guiding Device

For a long time, people have been inspired by the behavior of animals and plants, and have developed various bionic devices accordingly. Mosquitoes are one of the deadliest insects on the planet because they can transmit diseases through bites. In order to suck blood, the mosquito must penetrate its own tiny blood sucking probe through the skin to reach the blood vessel. Mosquitoes use a combination of mechanisms including insertion guides, allowing them to bite larger animals [155]. 

**Figure 12:**
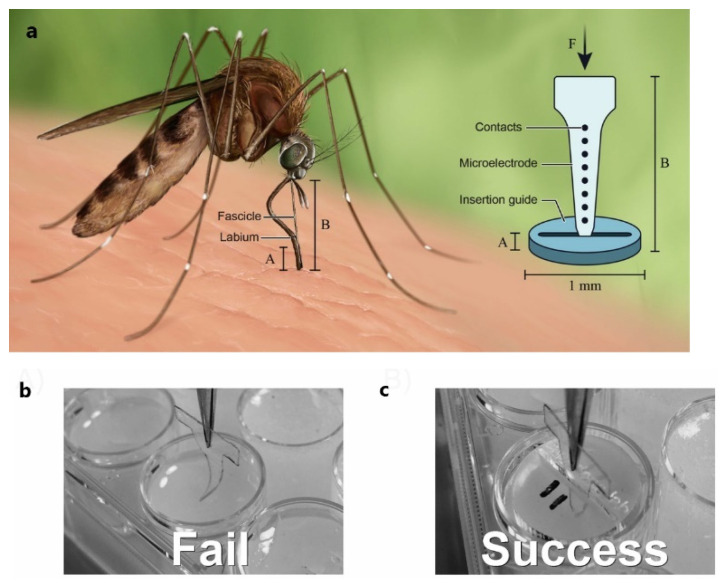
Boot device [155]: (**a**) Mosquito-inspired guide to reduce buckling of flexible microelectrodes during insertion into brain tissue. (**b**) Example of a failed insertion attempt without the guide in-place. Note the dummy microprobe buckling as it makes contact with the surface of the 0.6% agar model. (**c**) Example of a successful insertion with a guide in place.

Inspired by the guidance of the labia of mosquitoes, Shoffstall and co-workers [155] developed a new method of implanting flexible microprobes into the brain, as shown in Figure 12. Studies have shown that mosquitoes can penetrate the skin of the host with one needle, and the critical flexion force of each needle must be increased while reducing the force required to penetrate the skin. In order to prevent the fiber bundle from bending during implantation, the critical load must be higher than the penetration load. Euler’s formula by critical load is as follows:

(2) $$F_{Euler\;critical}=\frac{\pi^{2}EI}{{\left(KL\right)}^{2}}$$

In the formula, *E* is the Young’s modulus of the material, *I* is the moment of inertia of surface contact, *K* is the effective length coefficient, and *L* is the unsupported length of the column. By using the labia, the length of the load-bearing part of the fiber bundle can be effectively reduced, thereby increasing the critical buckling load. When the bionic guide is placed on the implantation site, the critical flexion force of the probe is increased by 3.8 times, which increases the success rate of probe implantation from 37.5% to 100%.

**Figure 13:**
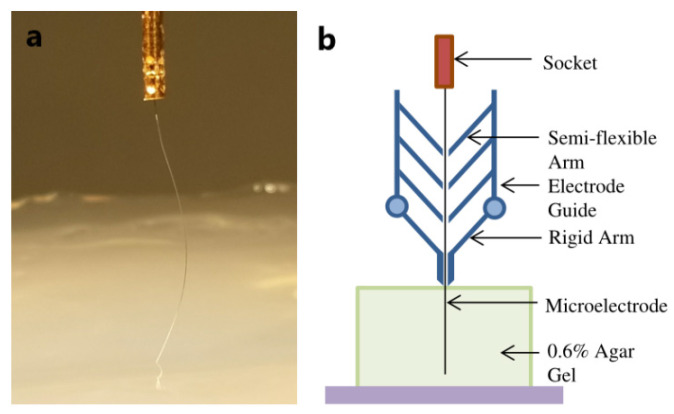
Boot device [150]: (**a**) buckling of Pt microelectrode during insertion in 0.6% agar gel and (**b**) proposed electrode guide.

In order to increase the critical flexion force during implantation, Muhammad A et al. [150] can be achieved by inserting a rotation and guiding device, as shown in Figure 13. For the implantation of cylindrical electrodes, CBF (critical buckling force) is calculated by Euler’s column formula, as follows:

(3) $$P_{cr}=\frac{K\pi^{3}Er^{4}}{4L^{2}}$$

In the formula, *P_cr_* is the CBF of the electrode (unit: Newton); *K* is the effective length coefficient (dimensionless), which depends on the end support of the electrode; *E* is the Young’s modulus of the material (unit: Pa); *L* is the effective length (Unit: meter); and *r* is the electrode radius (unit: meter). The *L* value can be significantly reduced by using a guiding device. This type of guide is different from traditional auxiliary implants, in that the guide is located above the brain, which avoids tissue damage caused by inserting the guide. The guide is composed of four pairs of separate arms, each pair of arms forms a micro-hole through which the electrode passes. The electrode can only be bent between two microholes, and the effective length of the bend depends on the ratio of spacing. This approach significantly increases CBF. At the same time, the rotation of the electrode probe can prevent the dura from sticking to the electrode and can maintain the stable implantation of the electrode. Based on the two strategies of guided insertion and rotation, they successfully implanted a 25 μm electrode into the rat brain 10 mm deep without bending.

By reducing the effective length of the implanted probe, the critical buckling load or critical buckling force of the probe can be increased, and the implant can be stabilized without using any other implantable auxiliary tools or hardeners. In addition, the cross-sectional area of the probe will not be increased during implantation, so as to avoid tissue damage during implantation.

## 5. Conclusions

As the most complex organ of the human body, the brain helps people obtain, process, transmit, and store information from the outside, and is the most important part of the human body. As a bridge linking the nerve-electrode interface, neural microelectrodes can help us better understand the brain. However, the traditional microwire electrode and silicon-based electrode are difficult to meet the compliance of human tissue due to their large Young’s modulus. The emergence of a flexible neural microelectrode is a good solution to the problem of modulus mismatch. Recent progress in flexible functional materials has enabled advances in flexible neural microelectrodes. Carbon-based materials, shape memory alloys, liquid metal, and other flexible organic electrode materials, such as conductive polymers have resulted in increasingly flexible microelectrodes, improved electrical characteristics, and increased biocompatibility of microelectrodes. Likewise, substrate materials, such as PDMS, PI, and LCP, have improved the flexibility and biocompatibility of microelectrodes. Electrode materials generally limit the mechanical compliance, but by integration with flexible substrates, they have improved mechanical performance while maintaining favorable electrical characteristics. In addition to advances in materials, the structural design of the neural microelectrode will also require extensive knowledge of neuroscience, from brain anatomy to neurophysiology. Brain-compatible interfaces have many hurdles before the technologies can be adapted for human subjects. This is mainly because of the complex structure of the human brain. Specifically, approaches such as the origami, kirigami, and mesh electronics mimic the soft tissue, integrating with cells. The ultrasmall carbon fiber microelectrode and microwire electrode can reduce the damage of the blood vessels and the local neuronal environment. The 2D/3D electrode array with flat and penetrating parts can record the electrical signals of neurons from brain surface (ECoG) and cortex (single unit action potential) well. However, flexible neural microelectrodes can be difficult to implant into the brain tissue. Therefore, this review introduces some implantation approaches of the flexible electrode probe into the brain tissue, such as using temporary coating, removable auxiliary equipment, choosing stimulus-responsive materials, applying a guiding device, and microfluidic drive. Although flexible neural microelectrodes are in the stage of rapid development, there are still many aspects to be improved. Therefore, the flexible neural microelectrode should pay attention to the following problems: (1) At present, most of the electrode implantation targets are rodents. Their brains are not as complex as those of primates. Experiments on some primates need to be sped up. (2) The recording and stimulation part of the front end of the implanted electrode is very small, which can meet the size of nerve cells well. However, the backend transmission and processing signal device is large, so how to realize the miniaturization of the backend device or wireless transmission is a problem that needs to be solved. (3) At present, the implantation time of an implantable neural microelectrode is mostly a few months or a year, and the long-term implantation of electrodes (within a few years) needs further improvement. (4) The miniaturization of the electrode implantation method is the key to avoid additional injuries, and the electrode implantation into minimally invasive surgery is a safer way. In short, the development of nerve electrodes has a long way to go.

## Figures and Tables

**Table 1:** A summary of the flexible electrode materials.

Electrode Material	Electrical Property	Young’s Modulus	Reference
PEDOT	1200 S·cm^−1^	2.6 ± 1.4 GPa	[44,45]
PT	10–100 S·cm^−1^	3 GPa	[37]
PPY	40–200 S·cm^−1^	430–800 MPa	[46,47]
PANI	5 S·cm^−1^	2–4 GPa	[46,48]
Graphene	243.5 ± 15.9 kΩ (∼200 µm diameter)	∼1 TPa	[41,49]
Carbon nanofiber (CNF)	∼1 MΩ (2 cm length, 25.7 × 16.6 µm^2^)	6–207 GPa	[50,51]
Glassy carbon	11.0 ± 5.4 kΩ (300 µm diameter)	20 GPa	[52,53]
Diamond	∼207.9 kΩ (0.0079 mm^2^)	∼103 GPa	[54]

**Table 2:** Doped electrode materials.

Raw Materials	Doping Material	Before Modification	Comparison before and after Modification	References
ITO microelectrode	coat PEDOT: PSS	High electrochemical impedance	Electrochemical impedance of the electrode is decreased, and the charge storage is increased.	[65]
Graphene fiber	Coat with platinum	Impedance value of GF-PC = 28.4 ± 4.1 MΩ·μm^2^, CSC = 200 ± 25 mC·cm^−2^	Impedance value of GF-Pt-PC = 3.9 ± 0.4 MΩ·μm^2^, CSC = 362 ± 45 mC·cm^−2^	[66]
PEDOT: PSS	rGO	CSC = 46.38 mC cm^−2^, Young’s modulus = 7.30 ± 0.50 GPa	CSC of PEDOT: PSS: rGO = 155.36 mC cm^−2^, Young’s modulus = 2–5 GPa	[64]
PEDOT	CNF	-	Impedance value of PEDOT: CNF = 1.28 MΩ·μm^2^ (1 kHz), charge injection limit = 10.03 mC cm^−2^	[67]
CF	B-CNW	CIC = 0.024 ± 0.008 mC·cm^−2^, impedance (1 kHz) = 133.4 ± 10.1 kΩ	B-CNW-CF CIC = 7.82 mC·cm^−2^, impedance (1 kHz) = 28.8 ± 4.2 kΩ	[68]
PPy	Au nano-particles, Dex	-	Effective surface area of the electrode is increased, resulting in a significant decrease in the impedance. Release of Dex anti-inflammatory drugs reduced astrocytes.	[69]

**Table 3:** Properties of polymeric substrates for neural implants.

Substrate Material	Young’s Modulus	Biocompatibility	Stability In Vivo	References
Parylene-C	2800 MPa	USP class VI	-	[83,84,85]
Polyimide (PI)	2300–8500 MPa	Yes	1091 days	[86,87]
SU-8	2870–4400 MPa	Mild reactivity	-	[86,88]
Polydimethylsiloxane (PDMS)	0.36–8.7 MPa	USP class VI	>18 weeks	[85]
Liquid crystal polymer (LCP)	10,600 MPa	USP class VI	2.5 years	[85]
PMMA	2000 MPa	Yes	3–6 months	[89]
PA	4750 MPa	Yes	1200 days	[15]

**Table 4:** Different types of extracellular electrical signals in the brain [92].

Signal Type	Electrode Placement
Electroencephalography (EEG)	Scalp
Electrocorticography (ECoG)	Cortical surface
Local field potential (LFP)	Brain
Multi-unit activity	Brain
Single-unit activity	Brain
